# Ketamine as a Treatment for Anorexia Nervosa: A Narrative Review

**DOI:** 10.3390/nu13114158

**Published:** 2021-11-20

**Authors:** Johanna Louise Keeler, Janet Treasure, Mario F. Juruena, Carol Kan, Hubertus Himmerich

**Affiliations:** 1 Section of Eating Disorders, Department of Psychological Medicine, Institute of Psychiatry, Psychology & Neuroscience, King’s College London, London SE5 8AF, UK; janet.treasure@kcl.ac.uk (J.T.); hubertus.himmerich@kcl.ac.uk (H.H.); 2 South London and Maudsley NHS Foundation Trust, Bethlem Royal Hospital, Monks Orchard Road, Beckenham BR3 3BX, UK; mario.juruena@kcl.ac.uk; 3 Centre for Affective Disorders, Department of Psychological Medicine, Institute of Psychiatry, Psychology & Neuroscience, King’s College London, London SE5 8AF, UK; 4 Eating Disorder Service, Central and North West London NHS Foundation Trust, 1 Nightingale Place, Kensington & Chelsea, London SW10 9NG, UK; carol.kan@nhs.net

**Keywords:** anorexia nervosa, atypical psychedelics, eating disorders, esketamine, ketamine, narrative review, severe-enduring, treatment

## Abstract

Anorexia nervosa (AN) is a highly complex disorder to treat, especially in severe and enduring cases. Whilst the precise aetiology of the disorder is uncertain, malnutrition and weight loss can contribute to reductions in grey and white matter of the brain, impairments in neuroplasticity and neurogenesis and difficulties with cognitive flexibility, memory and learning. Depression is highly comorbid in AN and may be a barrier to recovery. However, traditional antidepressants are often ineffective in alleviating depressive symptoms in underweight patients with AN. There is an urgent need for new treatment approaches for AN. This review gives a conceptual overview for the treatment of AN with ketamine. Ketamine has rapid antidepressant effects, which are hypothesised to occur via increases in glutamate, with sequelae including increased neuroplasticity, neurogenesis and synaptogenesis. This article provides an overview of the use of ketamine for common psychiatric comorbidities of AN and discusses particular safety concerns and side effects. Potential avenues for future research and specific methodological considerations are explored. Overall, there appears to be ample theoretical background, via several potential mechanisms, that warrant the exploration of ketamine as a treatment for adults with AN.

## 1. Introduction: An Overview of Ketamine

**Figure 1:**
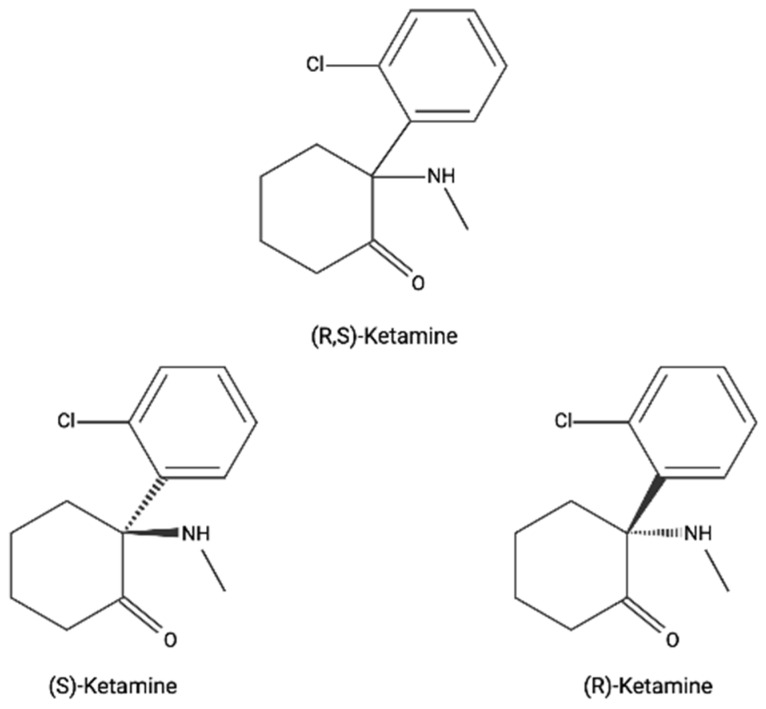
The molecular structure of ketamine, S-ketamine and R-ketamine.

Ketamine is an n-methyl-D-aspartate (NMDA) receptor antagonist that has traditionally been used for anaesthesia in larger doses [1]. It remains one of the two injectable anaesthetics under the World Health Organisation Model List of Essential Medicines, the other of which is propofol [2]. It is available in two enantiomers: the S(-) and racemic (R-) forms, referred to as esketamine and arketamine. However, when referred to as ketamine, this describes (R,S)-ketamine, which is a 1:1 racemic mixture of S- and R-ketamine enantiomers (see Figure 1 for the molecular structure). S-(Es)ketamine has an approximately four-fold higher affinity for the NMDA receptor site than R-ketamine and is three to four times as potent [3,4]. Moreover, esketamine generally produces fewer psychomimetic effects than R-ketamine. Ketamine can be administered via several routes that have varying bioavailability: intravenous (100%), intramuscular (90–95%), subcutaneous (90–95%), intranasal (30–50%), sublingual (20–30%), transdermal (10–50%) and oral (10–20%) [5]. Estimates of bioavailabilities increase when accounting for the contribution of norketamine; for example, in one study, the bioavailabilities of sublingual and oral ketamine increased from 32% to 54% and 23% to 59%, respectively [6]. Moreover, the area under the curve, peak plasma concentration and time to peak plasma concentration differ depending on the administration route and dosage (see Table 1).

Ketamine is rapidly metabolised (the half-life is 2–4 h for racemic ketamine and 5 h for esketamine [5]), mainly by the liver, and is excreted renally through urine and faeces. Its by-products include hydroxyketamine, norketamine, dehydronorketamine and six hydroxynorketamine metabolites [7]. The main metabolic pathway is N-demethylation to an active metabolite norketamine by CYP3A4. There is increasing interest in the metabolites of ketamine. For example, research has demonstrated the importance of the (2S,6S;2R,6R)-hydroxynorketamine metabolite for the antidepressant effects of (R,S)-ketamine [7], which is independent of NMDA receptor activity.

The first human dosage of ketamine was in 1964 at the University of Michigan, where it was titrated from a subanaesthetic dose to full anaesthesia [8]. Patient reports of the experiences included feeling “strange” and having “no feeling in their arms or legs” [8]. These experiences contributed to its classification as a “dissociative anaesthetic” by Domino and Corssen [4]. In 1970, ketamine was approved by the Food and Drug Administration (FDA) for use as an anaesthetic in children, adults and the elderly. Four years later, in 1974, ketamine was used for the first time within a psychiatric setting as an adjunct to psychotherapy for depression in Argentina [9]. Since then, advances in the field of ketamine have led to randomised controlled trials investigating its use for the treatment of various psychiatric disorders, including depression [10], anxiety disorders [11], alcohol and substance use disorder [12,13,14] and post-traumatic stress disorder [15]. Unfortunately, this research was somewhat obscured by ketamine’s classification as a class III substance in the US Controlled Substances Act due to it being popular as a recreational drug. However, in 2019, esketamine in intranasal form was licensed to treat treatment-resistant depression in the US and Europe by the FDA and European Medicines Agency (EMA), due to its rapid antidepressant effects.

Factors that contribute to the phenomenology of ketamine treatment include set (person factors) and setting (situational factors), terms introduced by Harvard psychologist Timothy Leary [16]. Set refers to the individual’s internal state, such as the psychological preparation and intentions for the experience, as well as intrinsic factors such as mood, psychopathology, personality, fears and wishes [17]. Setting refers to any environmental factors, including the physical, social and cultural environments [18]. Importantly, the nature of the set and setting determine the ketamine experience; optimising both factors is important to minimise the chances of an adverse psychological experience (i.e., a “bad trip”). Set can be optimised by providing patients with “sitters”, who help the patient prepare for the ketamine experience, set intentions, support them through the session and aid them in integrating its meaning afterwards. On the other hand, researchers and clinicians often optimise the setting by providing a calm and relaxing environment with minimal stimuli and bright light; an overly clinical or antiseptic environment may increase anxiety (for comprehensive guidelines, see [19]). Additionally, patients may be provided with eye shields and a carefully prepared music playlist and are guided to direct their attention inwards [20].

As aforementioned, the bioavailability of ketamine differs depending on the administration route, although it is unclear how or whether bioavailability is related to therapeutic effects [21]. Regardless, one study found dose-related antidepressant effects across all administration routes tested, including intravenous (IV), intramuscular (IM) and subcutaneous (SC) [22]. In this study, SC administration resulted in the fewest adverse events [22]. By comparison, IM and IV can be painful to administer, and intranasal ketamine can be uncomfortable if administered in the same nostril over 40 min [21]. Oral ketamine produces a bitter taste. However, oral, SC, IM and intranasal ketamine are more convenient than IV ketamine and can be used in everyday clinical care, as the latter requires an anaesthetist to be present for administration [21]. Overall, it is unclear which administration route is optimal within psychiatry, although within the context of treatment-resistant depression, intranasal esketamine alongside an antidepressant seems the most feasible and effective [5].

## 2. Materials and Methods

The purpose of this narrative review is to synthesise a theoretical rationale for the use of ketamine in the treatment of anorexia nervosa, integrating research across multiple disciplines (e.g., investigational research of ketamine for other psychiatric disorders). A literature search in the electronic databases MEDLINE and PubMed was performed until 15 September 2021, focusing on neurobiological models of anorexia nervosa and ketamine use within a psychiatric setting. An analysis of eligible publications using MeSH keywords such as “ketamine”, “neuroplasticity”, “anorexia nervosa” and “neurobiol” was conducted. Articles published in English were considered, and there were no restrictions on the type of article for inclusion; population-based studies, reviews, systematic reviews, meta-analyses, clinical trials and theoretical papers were included in this narrative review. Reference lists of publications were searched in order to identify additional eligible articles.

## 3. The Neurobiological and Psychological Effects of Ketamine

When administered at subanaesthetic doses, dissociative and mild psychological effects emerge within 15 min [23]. Psychological effects can include dissociation, alterations in perception, depersonalisation and derealisation, amongst others. These effects are transient and usually resolve within 90 min [24]. The antidepressant effects are rapid after the psychological effects dissipate, with effects on depressive symptoms persisting for a week in some patients [24]. This temporal sequence may indicate neuroadaptation to the effects of ketamine in the brain [25]. This aligns with studies revealing the positive influence of ketamine on neuroplasticity and, specifically, synaptogenesis.

Whilst ketamine is an NMDA receptor antagonist, its molecular action is more complex than a simple blockade of NMDA receptors. In fact, at low doses, ketamine paradoxically stimulates glutamate transmission [26]. This is achieved by both increased glutamate release and also increases in AMPA receptors at synapses, which are ionotropic transmembrane glutamate receptors [26]. This, in turn, causes increases in brain-derived neurotrophic factor (BDNF) release, which stimulates the mammalian target of rapamycin (mTOR) via Atk (protein kinase B) and ERK signalling. mTOR is a protein kinase that is important for the control of protein translation and contributes to long-lasting synaptic plasticity by increasing the number and density of synapses. Thus, the positive effect of ketamine on synaptogenesis is thought to reverse the reduced synaptic density that results from chronic stress and depression [27,28] and is implicated in its antidepressant effects.

As well as increasing synaptogenesis, rodent studies have indicated that ketamine increases neurogenesis and downregulates stress-induced inflammation. Adult neurogenesis refers to the birth of new neurons from stem cells, which occurs at a high rate in the hippocampus and is mediated by growth factors such as BDNF and vascular endothelial growth factor (VEGF). Studies in rodents have demonstrated increases in neurogenesis after ketamine administration both in vitro [29] and in vivo [30,31], which is thought to contribute to its sustained antidepressant effects [31]. Similarly, increases in BDNF and VEGF in the medial prefrontal cortex (mPFC) and hippocampus following ketamine administration are thought to contribute to this increase in neurogenesis [32].

Ketamine has also been shown to attenuate stress-induced inflammatory responses in rats. In one study, rodents exposed to chronic unpredictable mild stress exhibited greater hippocampal levels of proinflammatory cytokines (interleukin (IL)-1β, IL-6 and tumour necrosis factor (TNF)-α), which were reduced following a low dose of ketamine [33]. Notably, another study found decreases in proinflammatory cytokines in rodents exposed to stressors following ketamine administration but did not observe a reduction in corticosterone [34]. Thus, these effects may be independent of stress hormones. Whilst studies in humans are scarce, these findings have been replicated in patients with depression, with several studies finding decreases in various proinflammatory cytokines after ketamine treatment [35,36,37]. Whether the anti-inflammatory and antidepressant properties of subanaesthetic ketamine administration are related is debated, with some studies finding an association [36,37] and others finding no association [35,38]. Whilst more research is needed, it is possible that reductions in inflammation partially contribute to the therapeutic effects of ketamine.

## 4. Anorexia Nervosa

Anorexia nervosa (AN) has the highest mortality of any psychiatric disorder, with a standardised mortality rate of between 3.2 and 10.5 [39,40]. It is characterised by dietary restriction and weight loss behaviours (e.g., exercise), leading to significantly low weight. Prognosis is often poor, with estimates of relapse including 59% at nine years of illness [41] and 30% at 15 years of illness [42]. There are several biopsychosocial models that seek to explain the aetiology of AN, such as the Cognitive Interpersonal Maintenance Model [43]. Nevertheless, the neurobiological underpinnings of the disorder remain ambiguous. There are no approved psychopharmacological medications for AN, and the treatment options are otherwise limited [44]. Moreover, a large proportion of patients are treatment-resistant and therefore fail to gain weight. Treatment options are limited for this patient group [45], which has been described as a “crisis” in the field [46].

Psychiatric comorbidity is common in AN, even after the eating disorder has been resolved [47]. A study of 11,588 adults in eating disorder clinics in Sweden was conducted, with comorbidities including mood disorders (33–50%), generalised anxiety disorder (28–35%), social phobia (14–17%), obsessive-compulsive disorder (7–8%), post-traumatic stress disorder (PTSD; 3–7%) and substance use disorder (4–11%) [48]. Other data from separate geographical locations support similar prevalence rates [49,50,51,52], although other estimates of anxiety disorder comorbidity have been higher at ~55–85% of patients [53]. Features of autism spectrum disorder (ASD) are also highly prevalent in AN, with one study finding a 16.3% prevalence in a sample of 92 participants [54].

Patients with AN often report anhedonia, a lack of self-compassion, feelings of failure and suicidal ideation [49,55,56], and AN is associated with a higher risk of suicide [57]. Comorbid depression is linked to poor outcomes in patients, particularly those with severe-enduring AN (SE-AN), whereby patients with comorbid depression are six times more likely to remain unrecovered at a 22-year follow-up [58]. Individuals with AN often report that engaging in disordered eating behaviours allows them to cope with or avoid difficult emotions; affect regulation may be a maintaining factor for the disorder [59]. Additionally, anxiety is a feature within the syndrome of AN, with high levels of fear and anxiety around food, weight and body shape and stereotyped eating behaviours. In the majority of cases, symptoms of anxiety disorders (e.g., generalised anxiety disorder, social phobia and obsessive-compulsive disorder) precede the onset of AN [60,61]. Moreover, symptoms of anxiety as measured by the State-Trait Anxiety Inventory [62] often remain high even after recovery [63,64]. Another risk factor for the development of AN is childhood trauma, most of which are related to negative sexual experiences [65].

Genetic studies suggest overlapping aetiology between AN and some psychiatric comorbidities (e.g., depression and obsessive-compulsive disorder) [66,67,68]. However, psychopharmacological drugs often show little efficacy in terms of weight gain in AN; a meta-analysis found no benefit in the weight outcomes from both antipsychotics and antidepressants in comparison to the placebo [69]. New approaches to the management of comorbidities, such as depression, in AN are warranted, which may, in turn, alleviate eating disorder (ED) symptoms. The evidence for the use of ketamine in the treatment of each respective comorbidity will be addressed in the following sections.

## 5. The Use of Ketamine in Commonly Comorbid Psychiatric Disorders

### 5.1. Depression

Patients with treatment-resistant depression defined as those who have failed to achieve remission (or at least a 50% improvement in mood) after two antidepressants, are candidates for esketamine nasal spray therapy in conjunction with an antidepressant. Notably, the National Institute of Care Excellence (NICE) in the United Kingdom do not recommend nasal spray esketamine for depression on the grounds of poor cost-effectiveness [70]. This is currently being reviewed. In research, the most common administration route in clinical trials is intravenous (IV) ketamine [71]. Studies show that IV ketamine elicits a rapid antidepressant response in major depressive disorder and treatment-resistant depression, acting within 24 h and providing a response after a single IV administration of subanaesthetic doses (0.5 mg/kg) for 4–7 days [72]. There has been extensive research investigating ketamine as a treatment option for this population. For example, it has been found that a third of patients with treatment-resistant depression achieve remission [10], with higher rates of remission associated with repeated administrations. All symptoms of depression have been found to be reduced, including suicidality [25]. Additionally, improvements in aspects of memory and learning have been found in patients with depression after ketamine treatment, such as working memory and visual learning memory [73]. Studies of ketamine for depression have, for the most part, used adult samples, although a systematic review found reductions in depressive symptoms, suicidality and mood lability in adolescents given ketamine for treatment-resistant psychiatric conditions [74]. Thus, there is emerging evidence for its use in adolescents, although, notably, esketamine is only licensed for use in adult patients.

Whilst esketamine has fewer psychotomimetic effects than R-ketamine, it has been suggested that these psychotomimetic effects may increase the antidepressant efficacy [75,76]. Whilst considered a side effect, the psychotomimetic effects can have transformative psychological effects akin to those elicited from other psychedelic experiences (e.g., psilocybin [77,78]). Additionally, there have been concerns raised regarding the cognitive effects of ketamine, since it tends to produce impairments in cognitive function, although the majority of this evidence is in chronic users [79]. However, in acute doses, it appears that neurocognitive function is sustained or even improved [80]. More research should be conducted to ascertain the impact of therapeutic ketamine on cognition in patients with depression.

### 5.2. Obsessive-Compulsive Disorder

A systematic review of 11 studies of patients with OCD found that intravenous ketamine improves obsessive-compulsive symptomatology, with rapid effects that last from days to weeks [81]. Moreover, the effects of ketamine on OCD are prolonged if augmented with cognitive behavioural therapy (CBT). In an interesting case study of a patient with treatment-resistant depression, psychosis and OCD, oral esketamine was combined with deep brain stimulation targeting the ventral anterior limb of the internal capsule, demonstrating long-term benefits over 18 months [82]. Overall, there is a requirement for more research into the use of ketamine for OCD, although there is preliminary evidence for reductions in obsessive-compulsive symptomatology.

### 5.3. Autism Spectrum Disorder (ASD)

The evidence for using ketamine to treat the core symptoms of ASD (i.e., broadly, impairments in social interaction and restrictive/repetitive behaviours) is preliminary. One randomised controlled pilot study used two doses of intranasal ketamine and saline placebo in a crossover design on a population of young people with ASD [83]. There were no specific effects of ketamine on the clinician- and self-reported measures of autism; the rates went down both after the placebo and ketamine. Notably, ketamine was well-tolerated. However, a single case study found a dramatic reduction in the core symptoms of ASD for 36 h following a preoperative ketamine treatment [84]. Moreover, another single case study found improvements in depressive symptoms and the duration of eye fixation (an index of social skills) across 12 doses of intranasal ketamine in an individual with ASD and other psychiatric disorders [85]. Overall, the initial results were mixed, and further research is needed to ascertain whether ketamine is efficacious in addressing some of the core symptoms of ASD.

### 5.4. Anxiety Disorders

#### 5.4.1. Generalised Anxiety Disorder and Social Anxiety Disorder

Several studies over the last ~5 years have demonstrated the efficacy of ketamine for the treatment of anxiety disorders such as generalised anxiety disorder (GAD) and social anxiety disorder (SAD). For example, a double-blind RCT in patients with SAD found greater reductions in the symptoms of SAD in patients given IV ketamine than patients given a saline placebo [11]. A later double-blinded RCT in patients with SAD and GAD compared subcutaneous ketamine to a midazolam placebo, finding a dose-responsive (0.25, 0.5 and 1 mg/kg) reduction in anxiety in those given ketamine within an hour of dosing, which persisted for a week [86]. Following this, Glue and colleagues investigated the safety, tolerability and efficacy of extended-release oral ketamine tablets in seven responders from their RCT [87]. Over 96 h, participants showed reductions in self-reported measures of fear, anxiety and depression. However, it is important to note that the small sample size meant that no inferential statistical analyses could be conducted, and the results should be considered preliminary but promising. To our knowledge, no studies have examined intranasal esketamine for anxiety disorders, nor have they paired ketamine with adjunctive therapy, which is a potential area for future research.

#### 5.4.2. Post-Traumatic Stress Disorder

The fear response associated with a trauma-related stimulus may be targeted by ketamine. In addition, rodent models have demonstrated ketamine’s ability to promote fear extinction, which is suggested to occur via mTORC1 signalling [88]. Thus, it may be appropriate as an adjunct to extinction therapies, whereby a fear-associated cue is exposed to patients with the aim to form a new memory associated with it via inhibitory learning [89]. In this way, ketamine treatment may be especially efficacious for PTSD when “harnessing a window of ketamine-induced neuroplasticity” [90].

Early studies of ketamine for PTSD in humans administered the drug immediately after exposure to trauma, which produced mixed findings [91]. Later studies have investigated its use to treat chronic PTSD, and whilst, to date, there are few randomised-controlled trials (RCTs), the results are promising. For example, in one double-blinded RCT of 41 chronic PTSD patients, the PTSD symptoms and depression improved in response to ketamine as opposed to midazolam [15]. Another study found increases in the length of the clinical response to a mindfulness-based extinction and reconsolidation cognitive therapy in patients given IV ketamine relative to a saline placebo [92]. Overall, these preliminary findings were promising, and ketamine is likely a viable treatment option for patients who have experienced trauma.

### 5.5. Substance and Alcohol Use Disorders

There have been several reviews, including one systematic review, detailing the antiaddiction properties of ketamine [12,13,14]. Collectively, the studies have suggested that ketamine improves cravings, motivations to quit, physiological reactions to withdrawal and, importantly, reduces or completely abolishes the self-administration of drugs/alcohol [13]. For addiction, ketamine is often administered alongside psychotherapy, termed “ketamine psychedelic therapy” (KPT), which generally consists of a preparation phase, dosing phase and integration phase. This allows patients to fully take advantage of a hyperplastic brain state. Interestingly, this idea has been harnessed by a study investigating the potential for ketamine to rewrite the maladaptive reward memories associated with alcohol and drugs [93]. Participants were presented with a glass of beer and then retrieved maladaptive reward memories through exposure to a beer-related cue. Participants administered ketamine directly after this reported lower alcohol consumption over 10 days over and above participants who were either given the retrieval task or ketamine alone. The authors suggested that the ketamine facilitated the rewriting of drinking memories, which occurred within a critical reconsolidation window [93]. This brings into light the potential role of ketamine in enhancing the efficacy of therapies or abolishing learned associations via neuroplastic mechanisms.

## 6. Ketamine and the Neurobiology of Anorexia Nervosa

### 6.1. Neurotransmitters

#### 6.1.1. Serotonin

Serotonin, or 5-hydroxytryptamine (5-HT), is a monoamine neurotransmitter produced mainly in the gastrointestinal tract and also by the raphe nuclei, located in the brainstem. Thus, 5-HT has multifaceted functions and modulates food intake, body weight control, mood, cognition, learning and memory. There are many 5-HT receptors, ranging from 5-HT_1_ to 5-HT_7_, with a high density of receptors in the prefrontal cortex and hippocampus [94]. However, the 5-HT_1_ and 5-HT_2_ receptor families are of the most interest in the context of AN.

There is evidence for dysfunctions in the serotonin system in AN. For example, patients with AN show reduced levels of serotonin and markers of serotonin (e.g., tryptophan) in the acute stages, which normalise after recovery [95], together with depletions in 5-HT_1A_ and 5-HT_2A_ receptor densities [96]. However, some abnormalities persist after recovery. For example, 5-HT_2A_ receptor activity has been found to be abnormal in individuals recovered from AN [97]. Additionally, there have been several studies indicating genetic differences related to serotoninergic activity in AN (e.g., the S allele of the 5-HTTLPR gene [98,99]), one of which is a polymorphism of the 5-HT_2A_ receptor gene [100,101].

It is possible that the dietary restriction associated with AN leads to a depletion of the dietary supplies of tryptophan, which is an amino acid that is a chemical precursor to 5-HT. Alterations in serotonin signalling in limbic pathways (e.g., mesocorticolimbic pathway) and structures (e.g., the hippocampus, hypothalamus, amygdala and thalamus) may contribute to various features of AN, such as obsessiveness, body image distortions, low mood, anxiety, fear, dietary behaviour and their response to SSRIs [102].

Several studies have suggested that the antidepressant effects of ketamine are partially due to its effects on 5-HT, showing some similarities to traditional SSRIs. In vivo microdialysis studies in rodents given an acute subanaesthetic dosage of ketamine showed increases in 5-HT_ext_ in the mPFC [103]. Additionally, a study in nonhuman primates demonstrated a downregulation of selective 5-HT transporters (SERT) after an acute IV ketamine injection [104]. Overall, there is evidence for the importance of 5-HT_1A_ and 5-HT_1B_ receptor agonism for the antidepressant effects of ketamine (see [105] for a review). It is thought that this may occur as a downstream effect of hippocampal NMDA receptor inhibition and AMPA receptor activation. This may normalise the aforementioned alterations shown in AN and improve mood and/or comorbid depression.

#### 6.1.2. Dopamine

Dopamine is a neurotransmitter important in reward processing, produced mainly in the areas of the brain implicated in reward, such as the substantia nigra and ventral tegmental area. Reward constitutes three main processes: “liking” (a hedonic impact), “wanting” (incentive salience) and learning (habit formation and forming associations) [106]. Whilst dopamine is implicated in all reward processes, studies suggest that it is most important for “wanting” processes [107,108] and, thus, is a large feature in theories of addiction.

In AN, alterations in the dopaminergic reward system have been noted, although the findings are complex and often contradictory [109]. Overall, the findings suggest that alterations in dopamine in AN may drive difficulties in discriminating between punishment and reward, which is related to increased levels of anxiety and harm avoidance [110]. It has been suggested that AN-related cues and behaviours become rewarding over time, as increases in stress hormones attributable to food restriction can stimulate the dopamine reward system via the hypothalamic–pituitary–adrenal axis [111]. Repeated dopamine signalling may then aid in the transfer of these behaviours into habits [111,112]. Thus, it has been suggested that treatments focus on developing associations with recovery goals and egosyntonic aspects of the disorder rather than simply on food cues [110]. However, this is likely to be especially difficult in patients who remain underweight or have the treatment-resistant form of AN, as poor engagement in therapies and cognitive difficulties may interfere.

Ketamine has been found to modulate the brain circuits related to reward and motivation [113,114]. For example, resting-state functional magnetic resonance imaging (MRI) scans of patients with treatment-resistant depression two days post-ketamine infusion demonstrated increases in the frontostriatal connectivity in one study [115]. An additional study provided evidence for increases in synaptic plasticity in the hippocampus–accumbens pathway, which occurred partially due to the activation of D1 receptors [116]. Thus, it is possible that using the suggested psychological approaches above within a window of ketamine-induced neuroplasticity may instigate changes in reward functioning and reward-related associations with disordered cues.

### 6.2. Neuroplasticity and Neuromorphology

Neuroplasticity is regulated by the noradrenergic, serotoninergic, anticholinergic and glutamate systems [117,118]. Neuropsychological studies of AN indicate reductions in neuroplasticity, with decreased cognitive flexibility, memory and learning, which will be discussed in the following section. Malnutrition, chronic stress and the presence of psychiatric comorbidities can lead to reductions in BDNF and hippocampal volume, together with increases in proinflammatory cytokines. The presence of neuroinflammation has been noted in AN, with studies finding increases in proinflammatory cytokines (e.g., TNF-α, IL-6 and IL-1β [119,120]), some of which normalise after weight restoration (e.g., IL-6 [120,121]). Additionally, chronic psychosocial stress can alter the proinflammatory cytokine pathways [122,123]. Stressors include, but are not limited to, childhood adversities and life events, caregiver stress and loneliness, all factors that can be linked to the development of AN [124].

Stress-induced depression-like behaviours in rodents are associated with increases in proinflammatory cytokines but also with decreases in BDNF and neurogenesis [125,126,127]. Inflammation and aberrant concentrations of BDNF have been noted in several other psychiatric disorders that are often comorbid with AN, such as PTSD and depression [128,129,130,131,132]. Thus, comorbidities may contribute to neuroinflammation in AN. Similar to in depression, patients with AN show low levels of BDNF, which generally resolves following weight restoration [133].

In the acute stages of AN, global decreases in grey matter volume of 4% to 5% are observed [134,135], which are more apparent in certain structures such as the hippocampus [136]. This region is particularly vulnerable to long-term malnutrition due to its high levels of neurogenesis [137,138,139,140]. Additionally, a loss of white matter is observed [141], which is more pronounced in adolescents with AN, perhaps resultant from the high vulnerability of the brain during development [135]. Generally, the grey and white matter volumes increase following weight restoration, although they are not always fully normalised [141]. Notably, one study found that white matter volume loss at admission in adolescents was predictive of recovery at 1 year; the interruption of white matter tract development as a result of malnutrition may contribute to chronicity [142].

We have speculated in previous publications that the effects of chronic malnutrition and traumatic events and/or chronic social stress associated with living with AN may contribute to neuroinflammation and both impaired neuroplasticity and neurogenesis [136]. However, these speculations are yet to be confirmed with robust empirical research, and many questions remain. Whilst there is little research investigating neurogenesis in human patients with AN, a study using an activity-based anorexia (ABA) rodent model found decreases in cell proliferation in the dentate gyrus following three days of ABA [143].

In people with AN, many of these parameters often normalise following weight restoration and recovery. However, a proportion of patients fail to weight restore (i.e., are “treatment-resistant”) and thus experience persistent impairments in neuroplasticity, which is a likely barrier to successful treatment. Targeting neuroplasticity in treatment is likely to increase its success in both acute and chronic cases of AN.

As aforementioned, the antidepressant effects of ketamine appear to be mediated by an increased glutamate release [144], which has been linked to downward increases in BDNF, neurogenesis and synaptic plasticity. Relatedly, a structural MRI study demonstrated increases in the hippocampal volume following a single dose of ketamine, which was associated with the treatment response [145]. Additionally, ketamine may mitigate the effects of chronic stress on the brain [146], as well as associated increases in inflammatory markers [33]. Ketamine treatment has been shown to have positive effects on the neuropsychological parameters of memory and learning, which will be discussed in the following section.

### 6.3. Neuropsychology

Patients with AN often have difficulties in several aspects of cognition that may be linked to reduced neuroplasticity and hippocampal function, such as memory, learning and cognitive flexibility. Apparent deficits in memory in AN include overgeneralising autobiographical memories [147,148], poor immediate and delayed recall of story details [149], recall of locations [150] and pattern recognition memory [151]. Patients with AN have a negative bias when constructing future-directed thoughts compared to healthy controls [152], which may be related to a negative bias in memory retrieval [153]; it has been hypothesised that generating future-directed thoughts is reliant on the flexible recombination of events from the past [154]. Relatedly, adults with AN show selective impairments in aspects of cognitive flexibility such as task switching, which are also apparent in adults recovered from AN [155]. Whilst such depletions in cognition may not be at a threshold to be deemed an “impairment” [155], they may be precipitating or maintaining factors and are likely to contribute to the significant difficulty in engaging with psychological therapies.

Amongst and related to ketamine’s neurobiological actions, there are several qualities of ketamine that are likely to address the difficulties in several aspects of neuropsychology in AN. Ketamine has multifaceted effects on the memory [156], which is contingent on the dosage and length of use. For example, chronic ketamine abuse is associated with decrements in the episodic memory, working memory and semantic memory, the former two of which abate following drug cessation [156]. At subanaesthetic doses, ketamine has similar, albeit transient, effects. These effects have been harnessed in research examining whether ketamine as an adjunct to exposure therapy can be used to block the reconsolidation of trauma-related memories [157]. Reconsolidation refers to the process whereby the strength and course of existing memory traces are modified. Additionally, ketamine can facilitate extinction learning, which describes the process of a new memory being formed rather than an existing memory being modified; inhibitory learning is thought to antagonise the old memory [157]. Our team found preliminary evidence for generalised impairments in extinction learning in AN [158]. It is possible that ketamine may facilitate extinction learning against feared foods and food-related situations in patients with AN.

As aforementioned, individuals with AN often experience social anxiety [48]. Previous research has demonstrated that individuals with eating disorders have a higher vigilance for social signs of rejection and avoid social rewards [159]. Importantly, this sensitivity to interpersonal conflict and anxiety around social situations can interfere with the therapeutic bond and, thus, the outcomes of therapy. Ketamine has been reported to increase the social functioning of patients with depression, reducing rejection sensitivity, social avoidance, pessimistic thinking and a bias towards negative information, which facilitates a therapeutic bond [160].

Ketamine can induce alterations in bodily perceptions, including feelings of lightness and floating [161,162]. Patients with AN tend to report high levels of cognitive control and cognitive rigidity [163,164]. Patients also report that their eating disorders often feel intertwined with their identities [165] and report anguish at physically “taking up space” [166,167]. Additionally, disconnection from the self, others and the world is core to AN [168,169,170]. It is possible that ketamine may have an additional therapeutic impact for patients with AN by promoting flexibility, ego dissolution, detachment from one’s internal dialogue and openness to experiences [162]. Moreover, the mystical and spiritual experiences that often manifest during ketamine treatment [162] may enable patients to connect with their spirituality and alter their perceptions of both themselves in the context of the wider world and of the universe itself.

## 7. Ketamine as a Treatment for Anorexia Nervosa

### 7.1. Current Research

To date, there have been few studies investigating the therapeutic use of ketamine for AN, most of which have been case series or reports. Table 2 provides an overview of the study characteristics and main findings; all studies found reductions in the main outcome measures, including depression scores, suicidality and eating disorder psychopathology [171,172,173,174]. To date, all investigations of ketamine as a treatment for AN have been in adult patients. We would recommend that initial RCTs use SE-AN samples, who are typically adults due to the long duration of the illness as a criterion for inclusion and often have comorbid depression. However, given that ketamine has been found to alleviate depression in adolescents [74], future studies may endeavour to use it in a younger sample of patients with AN.

In all studies, no severe side effects were reported beyond those expected (e.g., transient headaches, mild nausea and sedation). However, as off-label, small and uncontrolled trials, these studies provided only preliminary evidence for its efficacy. To date, there have been no well-controlled feasibility studies using a large sample, although there is a pilot double-blinded crossover trial of oral ketamine versus midazolam registered on the Australian New Zealand Clinical Trials Registry (REG: ACTRN12618001393246p).

### 7.2. Side Effects and Safety Concerns

There are specific safety concerns when investigating pharmacological interventions for AN. Liver enzyme abnormalities, such as elevations in liver transaminases, are associated with a lower body mass index (BMI) and hypoglycaemia [175]. Repeated high doses of ketamine and prolonged ketamine abuse have been associated with hepatotoxicity and liver injury [176]. Whilst this causal pathway is not fully understood, it may be related to lipid peroxidation (oxidative damage) [177]. Liver function tests are therefore a necessary precursor to ketamine treatment in AN.

Moreover, upper and lower urinary tract dysfunctions are present in approximately 20–40% of recreational ketamine users [178,179,180], which tend to be discontinued following the cessation of ketamine use. There is a dose–response relationship between ketamine use and the probability of lower urinary tract symptoms, meaning that long-term usage may be a concern. This is a particular concern, as renal complications have been observed in patients with AN [181]. Thus, patients should be asked about polydipsia, haematuria, incontinence and pelvic pain; indicators of renal function in the blood (e.g., albumin to a creatinine ratio and glomerular filtration rate) should be monitored, and abnormalities would warrant the cessation of ketamine.

Cardiac complications/abnormalities are a notable feature of AN. For example, hypokaemia can be a consequence in patients who use self-induced vomiting as a compensatory behaviour to manage weight gain. This, in turn, can lead to prolonged QT intervals, markers of arrhythmias. Other cardiac complications include bradycardia/tachycardia, congestive heart failure and hypotension. Ketamine administration is associated with transient increases in the blood pressure and heart rate. The use of psychostimulants (e.g., amphetamines, methylphenidate, modafinil and armodafinil) should not be permitted, as they increase blood pressure. Moreover, patients on concomitant monoamine oxidase inhibitors (MAOIs) should have their blood pressure monitored closely, since MAOI usage has been reported to increase blood pressure [182]. Completing an ECG and measuring electrolytes prior to treatment may be recommended. Recent cardiovascular events or clinically significant cardiovascular conditions are also a specific contraindication of ketamine.

Other identified key side effects of ketamine include transient dissociative states, sedation, nausea and vomiting, dysgeusia (alterations in taste) and hypoesthesia (changes in touching sensation). Dissociation is more common in people with comorbid PTSD, which is likely due to it already being a feature of the disorder. Patients with comorbid PTSD should therefore be closely monitored, and studies should administer the Clinician Administered Dissociative States Scale (CADSS [183]) to investigate the incidence of dissociation in AN following ketamine treatment. The “setting” of the room should promote feelings of calm and relaxation, minimising bright lights and too many stimuli, and encouraging patients to focus on music and pleasant thoughts.

Psychosis may be a contraindication for ketamine; ketamine can induce psychotic episodes in people that have schizophrenia [184]. Sedation is more of a risk if patients are on opioids or benzodiazepines; thus, this should be considered when patients are screened for treatment. Nausea, vomiting and dysgeusia may be specific concerns in the context of AN, since they have the potential to be triggers. This will need to be examined closely in future research, although the aforementioned studies conducted so far have not indicated any of the above to be particular concerns.

## 8. Future Perspectives

AN is a particularly hard-to-treat population, and pharmacological trials in AN often suffer from a high dropout rate. People with AN have reported specific concerns around pharmacological interventions, such as fears around drug-instigating weight gain [185]. However, patients also report wanting medication to alleviate anxiety, eating disorder thoughts, poor concentration and sleep problems [185]. A recent survey conducted on 200 participants with eating disorders (*n* = 105 with AN) investigated views on psychedelic drugs as a treatment for eating disorders [186]. Approximately half of the participants expressed interest in participating in psychedelic interventional research in the context of various concerns. The concerns were mitigated when the participants were informed that esketamine was licensed for the treatment of treatment-resistant depression. Importantly, this survey highlighted important methodological considerations, including the need for collaboration with service users and those with lived experience in the design of trials. The participants emphasised the need for a safe, professional and controlled environment during the dosing, a good rapport with the research team, trust in the trial and trial team and the provision of psychoeducation about psychedelic drugs. They also emphasised the need for psychological preparation before the session and an assessment of the “set”. Importantly, a third of the overall group reported that they would never take part in such research. Thus, establishing an ongoing dialogue with service users will be an essential consideration in the design of clinical trials.

Overall, it is apparent that future studies should first aim to establish a safety profile of ketamine for AN through well-controlled feasibility and pilot studies. The outcome measures should be agreed upon with service users. Investigational studies of neuromodulation (e.g., transcranial magnetic stimulation and deep brain stimulation) in severe-enduring AN have found proximal improvements in depression, with improvements in eating disorder psychopathology and weight taking 12–18 months to emerge [187,188,189]. Therefore, whilst the BMI is often the metric of success in interventional trials in AN, for patients with severe-enduring AN where weight gain is slow, other metrics of improvement such as depression scores, social connectedness and quality of life should be considered, and long follow-up periods should be implemented. Given the hypothesised role of ketamine in providing a window of neuroplasticity, it will be essential that ketamine is combined with psychotherapy in future trials. Whilst the antidepressant effects of ketamine are transient, there is emerging evidence that psychotherapies (such as CBT) can extend the duration of antidepressant effects [190]. Additionally, the experiential components of ketamine may facilitate the therapeutic bond between patient and clinician [160], which is known to be a predictor of response to treatments [191].

Ketamine-assisted therapy generally follows a three-stage process: (1) preparation, where the patient and client discuss the mindset going into the experience, treatment goals and expectations and set intentions; (2) dosing, where patients are given the ketamine administration; and (3) integration, where the ketamine experience is reflected upon, and insights/lessons gained during the experience are integrated into the self. In the treatment of AN, it may be useful to combine specific therapeutic strategies with ketamine, such as compassion-based meditation exercises, yoga, cognitive bias modifications and exposure therapies. Similarly, specific therapeutic approaches may work well with ketamine, such as compassion-focused therapy, the Maudsley Model of Anorexia Treatment for Adults (MANTRA), family-based interventions and other psychotherapies. However, it is important to note that a “one size fits all” approach is unlikely to be successful, and treatments should be tailored to the individual.

## 9. Conclusions

The current treatment options for AN show limited efficacy, and addressing severe-enduring cases presents a particular challenge to clinicians and researchers. This review gave an overview of a conceptual rationale for the use of ketamine in the treatment of AN. Ketamine has proven to be an effective treatment for a range of psychiatric disorders, including depression, anxiety disorders, addiction and PTSD. Relatedly, it was recently licensed for treatment-resistant depression as a nasal spray esketamine device. In AN, there are several compelling reasons for its application, which were discussed. Ketamine may promote neuroplasticity, neurogenesis and hippocampal volume, and mitigate neuroinflammation. As a rapid antidepressant, ketamine may also reduce depression in patients with AN, which may be a barrier to treatment. In combination with therapies, it may also promote cognitive flexibility, openness, open-mindedness and detachment from the self whilst also promoting a therapeutic bond. However, there are specific safety concerns that require consideration in the treatment of AN with ketamine, and future studies designed in collaboration with service users are warranted in order to establish its efficacy, acceptability and safety.

## Figures and Tables

**Table 1:** Single administration of ketamine: selected pharmacokinetic parameters.

Parameter	Administration Route		
	Intravenous	Sublingual	Oral
Dosage (mg)	10	25	25
C_max_ (μg/L) M ± SD	156 ± 161	28.6 ± 6.6	22.8 ± 12.8
T_max_ (h) M ± SD	0.24 ± 0.29	0.76 ± 0.51	0.96 ± 0.8
AUC/dose (μg.h/L.mg) M ± SD	13.4 ± 2.4	4.0 ± 1.9	3.1 ± 0.7

Notes: AUC = area under the curve, Cmax = peak plasma concentration, M = mean, SD = standard deviation and tmax = time to peak plasma concentration (adapted from [6]).

**Table 2:** Characteristics and main findings of studies of ketamine as a treatment for patients with eating disorders.

Study [Ref]	Study Design	N	Diagnosis	Administration Route	Dosage	Main Findings
Dechant et al. [171]	Case study	1	SE-AN and MDD	IV R-Ketamine	9 × 0.5 mg/kg over 40 min	Reduction in depression and suicidality.
Mills et al. [172]	Case series	15	SE-AN	IV Ketamine	2–15 × 20 mg/h for 10 h	9/15 responded to treatment, with reductions in depression. and compulsive starving/eating.
Schwartz et al. [173]	Case series	4	SE-ED and TRD	IM/IV Ketamine	5–9 × 0.4–0.5 mg/kg	Improvements in depression, anxiety and eating disorder psychopathology over approx. days.
Scolnick et al. [174]	Case study	1	SE-AN and MDD	IV R-Ketamine	4 × 0.75 mg/kg over 40 min	Reduction in “anorexic voice” and depression and full and sustained remission.

Notes: IM = intramuscular, IV = intravenous, MDD = major depressive disorder, N = number, SE-AN = severe-enduring anorexia nervosa, SE-ED = severe-enduring eating disorders and TRD = treatment-resistant depression.
